# Cardiovascular Risk in Rheumatic Patients Treated with JAK Inhibitors: The Role of Traditional and Emerging Biomarkers in a Pilot Study

**DOI:** 10.3390/jcm14155433

**Published:** 2025-08-01

**Authors:** Diana Popescu, Minerva Codruta Badescu, Elena Rezus, Daniela Maria Tanase, Anca Ouatu, Nicoleta Dima, Oana-Nicoleta Buliga-Finis, Evelina Maria Gosav, Damiana Costin, Ciprian Rezus

**Affiliations:** 1 Department of Internal Medicine, “Grigore T. Popa” University of Medicine and Pharmacy, 16 University Street, 700115 Iasi, Romania; popescu.diana@umfiasi.ro (D.P.); daniela.tanase@umfiasi.ro (D.M.T.); anca.ouatu@umfiasi.ro (A.O.); nicoleta.dima@umfiasi.ro (N.D.); oana-nicoleta.buliga-finis@umfiasi.ro (O.-N.B.-F.); evelina.maria.gosav@umfiasi.ro (E.M.G.); ciprian.rezus@umfiasi.ro (C.R.); 2 IIIrd Internal Medicine Clinic, “Saint Spiridon” County Emergency Clinical Hospital, 700111 Iasi, Romania; 3 Department of Rheumatology and Physiotherapy, “Grigore T. Popa” University of Medicine and Pharmacy, 16 University Street, 700115 Iasi, Romania; elena.rezus@umfiasi.ro (E.R.); damiana.costin@umfiasi.ro (D.C.); 4 Rheumatology Clinic, Clinical Rehabilitation Hospital, 700661 Iasi, Romania

**Keywords:** rheumatic disease, cardiovascular risk, dyslipidemia, Janus kinase inhibitors, rheumatoid arthritis, lipoprotein(a), LDL cholesterol, triglycerides

## Abstract

**Background:** Despite therapeutic advances, morbidity and mortality remain high in patients with rheumatoid arthritis (RA) and psoriatic arthritis (PsA), primarily due to increased cardiovascular risk. **Objectives:** Our study aimed to evaluate the cardiovascular risk profile and biomarker dynamics in patients with RA and PsA treated with Janus kinase inhibitors (JAKis). To our knowledge, this is the first study assessing Lp(a) levels in this context. **Methods:** This prospective, observational study assessed 48 adult patients. The follow-up period was 12 months. Traditional cardiovascular risk factors and biological markers, including lipid profile, lipoprotein(a) [Lp(a)], and uric acid (UA), were assessed at baseline and follow-up. Correlations between JAKi therapy, lipid profile changes, and cardiovascular risk factors were investigated. Cox regression analysis was used to identify predictors of non-major cardiovascular events. **Results:** A strong positive correlation was observed between baseline and 12-month Lp(a) levels (r = 0.926), despite minor statistical shifts. No major cardiovascular events occurred during follow-up; however, 47.9% of patients experienced non-major cardiovascular events (e.g., uncontrolled arterial hypertension, exertional angina, and new-onset arrhythmias). Active smoking [hazard ratio (HR) 9.853, *p* = 0.005], obesity (HR 3.7460, *p* = 0.050), and arterial hypertension (HR 1.219, *p* = 0.021) were independent predictors of these events. UA (HR 1.515, *p* = 0.040) and total cholesterol (TC) (HR 1.019, *p* = 0.034) were significant biochemical predictors as well. Elevated baseline Lp(a) combined with these factors was associated with an increased event rate, particularly after age 60. **Conclusions:** Traditional cardiovascular risk factors remain highly prevalent and predictive, underscoring the need for comprehensive cardiovascular risk management. Lp(a) remained stable and may serve as a complementary biomarker for risk stratification in JAKi-treated patients.

## 1. Introduction

Rheumatoid arthritis (RA) and psoriatic arthritis (PsA) are chronic autoimmune rheumatic diseases characterized by persistent inflammation and progressive joint damage. These conditions predominantly affect women and are typically diagnosed after the age of 50 [1,2].

Beyond joint inflammation and disability, patients with rheumatic diseases face an increased risk of cardiovascular events, leading to higher mortality rates. The relative risk of cardiovascular events in these patients is nearly twice that of age- and sex-matched individuals without rheumatic diseases [3,4,5]. This risk is largely due to accelerated atherosclerosis, driven by both systemic inflammation and traditional cardiovascular risk factors such as dyslipidemia, arterial hypertension, diabetes mellitus (DM), smoking, and obesity [6,7].

Despite improvements in disease control, cardiovascular risk remains insufficiently recognized and addressed. Conventional risk prediction tools—such as SCORE (Systemic Coronary Risk Estimation) or QRISK (Research Cardiovascular Risk Calculator)—tend to underestimate cardiovascular risk in patients with systemic chronic inflammation [8,9,10]. To address this, the European Alliance of Associations for Rheumatology (EULAR) recommends multiplying the estimated cardiovascular risk by 1.5 in RA patients with long-standing active disease, positive rheumatoid factor (RF), anti-citrullinated protein antibodies (ACPAs), or extra-articular manifestation, to better reflect their true risk [11]. However, this adjustment is imprecise and does not account for inflammatory or lipid-related biomarkers, hence the need for more accurate and disease-specific risk stratification tools.

Assessing the impact of disease-modifying antirheumatic drugs (DMARDs) on cardiovascular risk is a very active area of research, partly motivated by the wide variability of the results obtained so far. Some studies suggest that targeting inflammatory cytokines with biologics can reduce cardiovascular event rates [12,13], while others report neutral or even adverse effects on cardiovascular risk, depending on patient factors and drug type [14,15,16].

Janus kinase inhibitors (JAKis), including tofacitinib, baricitinib, and Upadacitinib, have emerged as effective oral therapies for RA and PsA, particularly in patients with inadequate responses to conventional (csDMARDs) or biological DMARDs (bDMARDs) [17,18,19,20]. By selectively targeting the JAK-STAT pathway, JAKis reduce disease activity and improve physical function and patient-reported outcomes [21]. However, JAKis have been associated with certain side effects, notably an increased risk of thromboembolism and dyslipidemia, especially in older patients or those with pre-existing cardiovascular comorbidities [22].

One known effect of JAKi therapy is the alteration of lipid metabolism. By suppressing inflammation, via interleukin-6 (IL-6) reduction, JAKi can lead to increases in total cholesterol (TC) and low-density lipoprotein cholesterol (LDL-C) levels [23]. It remains unclear whether these lipid changes directly translate into higher cardiovascular event rates, but patients with pre-existing cardiovascular risk factors may require closer monitoring and intensive management of dyslipidemia during JAKi treatment [24,25]. Interpreting lipid changes in the context of active rheumatic disease is complex. Active RA can paradoxically be associated with lower TC and LDL-C despite heightened cardiovascular risk (the “lipid paradox”) [26,27,28]. Conversely, effective anti-inflammatory treatment often raises lipid levels without a proportional increase in cardiovascular risk [26,27]. RA patients may exhibit significant atherosclerosis (e.g., high coronary artery calcium scores) even when LDL-C is low, suggesting that conventional lipid measures may underestimate this risk [28]. Other markers, such as remnant cholesterol and triglycerides, have been linked to cardiovascular events in patients with RA and may better reflect risk amid chronic inflammation [27,29,30]. Lipoprotein(a) [Lp(a)], largely genetically determined and not substantially modified by standard lipid-lowering therapy, is another important risk factor for atherosclerotic disease. Elevated Lp(a) levels are associated with increased rates of acute cardiovascular events in both the general population and in RA patients [31,32]. However, the predictive value of Lp(a) in autoimmune rheumatic patients remains uncertain due to limited data.

The aim of our study was to evaluate the overall effect of JAKi on cardiovascular risk factors in patients with autoimmune rheumatic diseases, namely RA and PsA. We focused on assessing the prevalence and trend of traditional cardiovascular risk factors in JAKi-treated patients. Then, we sought to determine whether Lp(a) levels are influenced by JAKi therapy and whether elevated Lp(a) is associated with adverse cardiovascular outcomes. By using regression models, we attempted to identify clinical and biochemical predictors of non-fatal cardiovascular events.

## 2. Materials and Methods

### 2.1. Study Design and Ethical Approval

We conducted a prospective, observational, single-center study between September 2022 and June 2024 at the “Saint Spiridon” County Clinical Emergency Hospital and affiliated institutions in Iasi, Romania. All patients provided written informed consent before enrolling in the study. The study was approved by the Ethics Committees of the “Grigore T. Popa” University of Medicine and Pharmacy Iasi, the “Saint Spiridon” County Clinical Emergency Hospital Iasi, and the Clinical Rehabilitation Hospital Iasi. The study was conducted in accordance with the Declaration of Helsinki.

### 2.2. Cohort Selection

**Figure 1:**
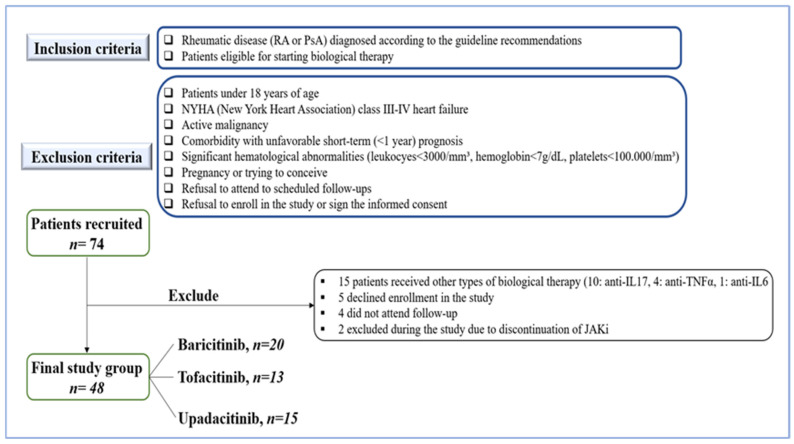
Flow diagram illustrating the inclusion and exclusion criteria, and the structure of the analyzed group according to JAKi. Legend: IL—interleukin; TNF—tumor necrosis factor.

Eligible participants were adults (≥18 years) with a confirmed diagnosis of rheumatic disease according to the EULAR criteria for RA or the CASPAR (Classification Criteria for Psoriatic ARthritis) criteria for PsA. Patients with prior cardiovascular events were allowed to enroll. A total of 74 patients met the eligibility criteria for treatment with JAKi. Of them, 48 patients completed 12 months of follow-up and were included in the final analysis. The inclusion and exclusion criteria are illustrated in Figure 1. One third of patients eligible for JAKi treatment were excluded for various reasons, such as assignment to a different biologic therapy by the treating physician (15 patients), refusal to enroll in the study (5 patients), failure to attend scheduled assessments (4 patients), and discontinuation of JAKi treatment (2 patients). One patient discontinued JAKi treatment due to pregnancy, and the other due to an acute illness (severe anemia and acute kidney injury), considered unrelated to JAKi therapy.

### 2.3. Clinical and Paraclinical Assessment

All patients underwent extensive clinical and paraclinical evaluation before initiating JAKi treatment (baseline assessment).

Patients’ demographic and epidemiological characteristics, traditional cardiovascular risk factors, medical history and concomitant medication were collected at baseline. Key demographic and epidemiological parameters included age, sex, smoking status, and body mass index (BMI). BMI was calculated using the weight-to-height ratio (kg/m^2^). The normal range was defined as 18.5–25 kg/m^2^. The comorbidities of interest were arterial hypertension, dyslipidemia, DM, and peripheral arterial disease (PAD). History of major cardiovascular events [e.g., myocardial infarction (MI) or stroke] was also noted. Among drugs, statins and conventional synthetic DMARDs (csDMARDs) were of special interest. Disease characteristics included the presence of RF and ACPA.

All patients underwent a comprehensive baseline physical examination, which included anthropometric measurements, heart rate, blood pressure (BP), and evaluation of peripheral pulses and heart murmurs. High BP was defined according to the current ESC/ESH Clinical Practice Guidelines for the Management of Arterial Hypertension as systolic BP ≥ 140 mmHg and/or diastolic BP ≥ 90 mmHg [33].

Paraclinical assessment involved blood sampling to evaluate hematological and biochemical parameters. A complete blood count was performed and neutrophil-to-lymphocyte ratio (NLR) was calculated (normal range 1–2). Lipid profile [TC, LDL-C, triglycerides, and Lp(a)], and uric acid (UA) were also assessed. Hyperuricemia was defined as UA > 6.2 mg/dL. Dyslipidemia was diagnosed if any of the following were present: (1) high TC (>200 mg/dL); (2) high LDL-C (>130 mg/dL); (3) high triglycerides (>150 mg/dL); or (4) high Lp(a) (>75 nmol/L). Since previous studies have shown that low to moderate dose of statins with moderate intensity are expected to reduce LDL-C by 30% [34], a significant decrease in LDL-C was defined as a reduction of ≥30%.

Cardiovascular evaluation included a standard 12-lead resting electrocardiogram (ECG) and transthoracic echocardiography (TTE). Scheduled ECG was performed at both baseline and 12-month follow-up, with the aim of identifying myocardial ischemia, conduction, or rhythm abnormalities. Scheduled TTE was performed in all patients at baseline and at 12-month follow-up, with a focus on left ventricular systolic function parameters, with the aim of identifying myocardial ischemia and potential contraindications for biological therapy. Unscheduled ECGs and TTE, performed when patients presented with new-onset or aggravated cardiovascular signs and symptoms, were also analyzed.

Clinical and paraclinical assessments performed at the 12-month follow-up after initiation of JAKi therapy mirrored those performed at baseline.

At the two key time points of the study (baseline, 12-month follow-up), all patients were evaluated by the same medical team, consisting of a PhD student (internal medicine specialist) and the supervising physician (PhD, senior in internal medicine). The TTEs were performed by a single physician, using the same equipment, ensuring consistency of measurements.

### 2.4. Statistical Analysis

A descriptive analysis was performed for all variables. Qualitative data were expressed as absolute values and percentages. Quantitative data were analyzed using the Statistical Package for Social Sciences (SPSS) software (version 23.0, Chicago, IL, USA). Continuous variables were presented as means ± standard deviation (SD) or medians and interquartile ranges (IQRs) depending on distribution. Normality was tested using the Kolmogorov–Smirnov test.

Categorical variables were compared using the Chi-square test. Correlation analyses between baseline and follow-up values were performed using Spearman’s correlation. Binomial logistic regression was used to explore associations between biological variables and the presence of dyslipidemia. Independent variables included Lp(a), LDL-C, triglycerides, TC, and UA.

Cox proportional hazard regression models were constructed to assess the relationship between clinical or biological variables and the occurrence of cardiovascular events.

For all statistical tests, *p*-values ≤ 0.05 were considered statistically significant.

## 3. Results

### 3.1. Study Characteristics, Clinical and Paraclinical Assessments

A total of 48 patients with autoimmune rheumatic diseases receiving JAKi therapy were included in the final analysis. Of these, 46 patients had RA and 2 had PsA. Most patients were women (83.3%) and overweight (75%). Among comorbidities, arterial hypertension was the most prevalent (58%), followed by dyslipidemia (35.4%). Twelve patients (25%) were already on lipid-lowering therapy. Baseline demographic characteristics, cardiovascular risk factors, comorbidities, and treatment profiles are summarized in Table 1.

Arterial hypertension and dyslipidemia followed a normal distribution, whereas other variables showed abnormal distributions, though none reached statistical significance.

For all variables that presented a non-parametric distribution, Spearman’s correlation coefficient was applied. The analysis revealed a moderate correlation between smoking and stroke history, and between age and arterial hypertension. A low correlation was observed between BMI and dyslipidemia or arterial hypertension. Notably, dyslipidemia showed a moderate positive correlation with statin use, arterial hypertension, DM, and age. A moderate inverse correlation was observed with JAKi therapy.

As illustrated in Table 1, patients were divided into three groups based on the type of JAKi they received. JAKi selection was influenced by patients’ comorbidities and personal preferences following shared decision-making. A total of 38 patients (78%) received concomitant csDMARDs, with the most commonly used being methotrexate (*n* = 21), followed by leflunomide (*n* = 16), while 10 patients were on JAKi monotherapy.

Baseline lipid profile results (T0) showed that approximately half of the patients had elevated TC (56%) and LDL-C (56%) values. Most of them also had an increased BMI. After one year of JAKi therapy (T1), these values showed a slight reduction, although therapeutic targets recommended by the guidelines were not reached. No patient recorded a drop below the normal range for these two parameters. In contrast, elevated triglyceride levels were observed in 12% of patients at T0, and increased to 17% at T1. However, this change was not statistically significant. The prevalence of elevated Lp(a) remained unchanged (19% at baseline versus 17% at 12-month follow-up).

Correlation analysis revealed a strong intra-individual correlation between baseline (T0) and follow-up (T1) Lp(a) levels (r = 0.926), and a moderate correlation for LDL-C (r = −0.322) and triglyceride levels (r = −0.258) between the two time points.

At the baseline evaluation, 7 of the 48 patients (14.6%) had ECG changes (ST-segment depression or T wave abnormalities) of chronic myocardial ischemia. No patient had a history of MI. During the follow-up period, three of these patients reported typical anginal symptoms, yet without evidence (ECG/TTE/biomarker) of acute ischemia. No patient required hospitalization for any type of acute coronary syndrome (ACS).

### 3.2. Biological Assessment and Correlations with Cardiovascular Risk Factors

To robustly test the hypothesis of Lp(a) stability under JAKi therapy, we performed multiple paired statistical analyses on the available dataset. Because Lp(a) followed a non-normal distribution at both time points, non-parametric methods were prioritized. Paired analyses demonstrated a statistically significant shift in Lp(a) values over time. A paired t-test showed t = 3.356, *p* = 0.0016, and the Wilcoxon signed-rank test confirmed the difference (W = 161.5, *p* < 0.0001). Repeated-measures ANOVA yielded similar results (F = 11.27, *p* = 0.0016).

To evaluate the potential role of Lp(a) as a predictive biomarker for cardiovascular events, we applied multivariate logistic regression models on our dataset. We used baseline Lp(a) levels as a predictor for the occurrence of events recorded at 12-month follow-up. The outcomes analyzed are summarized in Table 2. No major adverse cardiovascular events (MACEs), such as MI, stroke, thromboembolism, or cardiovascular deaths were reported. However, several non-major cardiovascular events were recorded, allowing for an exploratory Cox regression analysis. These events, although less severe, are clinically relevant and may signal early cardiovascular damage in high-risk patients.

During the 12-month follow-up, a total of 23 non-major cardiovascular events were recorded in 23 patients (47.9%), as detailed in Table 2. These events included elevated BP requiring therapeutic adjustments, anginal pain on exertion, without criteria for ACS, occurring in patients with a history of chronic coronary syndrome, and new-onset arrythmias detected on follow-up ECG.

Cox proportional hazard regression model was used to explore associations between baseline clinical parameters and time to first occurrence of a non-major cardiovascular event (as defined in Table 2). The model did not assess MACE or cardiovascular mortality because no such event was recorded during the study.

**Figure 2:**
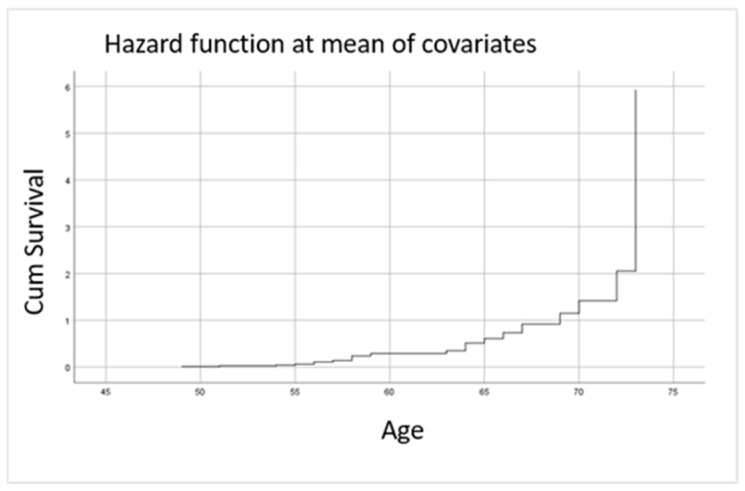
Estimated hazard function for the occurrence of first non-major cardiovascular event during follow-up, stratified by age. Legend: the function indicates an increasing trend in event probability after the age of 60. No MACE or deaths were observed during the study period.

Univariate analysis revealed that several traditional cardiovascular risk factors were associated with time-to-event outcomes during follow-up. Increased BMI, active smoking, and arterial hypertension were identified as predictors of non-major cardiovascular events, based on abnormal baseline Lp(a) levels (Table 3). Hazard function analysis showed that in patients with rheumatic diseases who also have these cardiovascular risk factors, the rate of non-major cardiovascular events increased exponentially after the age of 60 (Figure 2). Of note, BMI showed only a borderline significant association; thus, this result should be interpreted with caution.

**Figure 3:**
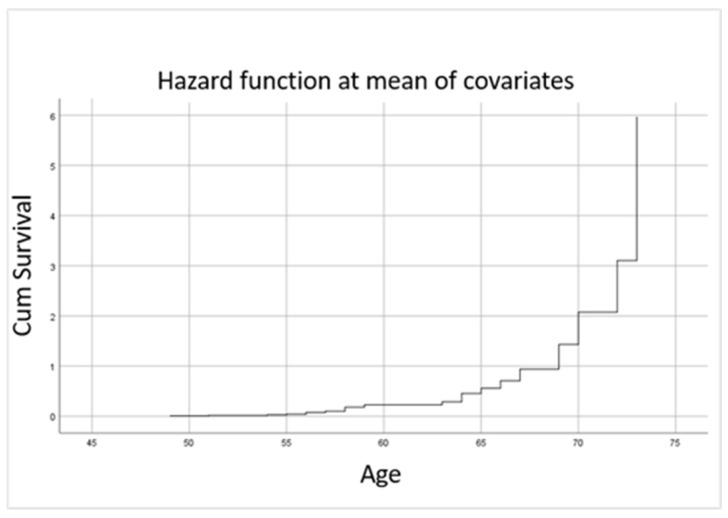
Age-related hazard function for non-major cardiovascular events in relation to UA and TC levels.

Univariate analysis of biological parameters showed that increased values of UA and TC were associated with an increased hazard ratio for non-major cardiovascular events (Table 4 and Figure 3).

Logistic regression analysis revealed no significant association between 12-month Lp(a) values and the incidence of non-major cardiovascular events over the one-year observation period.

## 4. Discussion

The results of this pilot study provide important insights into the cardiovascular profile of patients with RA and PsA receiving JAKi. It showed that one year of JAKi treatment was associated with the persistence of an atherogenic lipid profile and a high burden of traditional cardiovascular risk factors. Approximately half of the patients had dyslipidemia at baseline, and this proportion remained largely unchanged after 12 months of JAKi, indicating that lipid levels (TC, LDL-C, and triglycerides) did not improve and, in some cases, even worsened during the treatment period. Baseline risk factors—particularly obesity, active smoking, and arterial hypertension—were strongly associated with an increased rate of non-major cardiovascular events during follow-up, consistent with known risk patterns in rheumatic disease populations [35,36]. The high prevalence of obesity (75%) and hypertension (58%) in our cohort reflects the well-recognized association of these comorbidities with inflammatory rheumatic diseases. Obesity can exacerbate disease activity and increase comorbidities in RA/PsA [37,38], while chronic inflammation contributes to hypertension by inducing vascular stiffness and endothelial dysfunction [39,40]. Importantly, our Cox regression analyses reinforce the central role of traditional cardiovascular risk factors in predicting non-major cardiovascular events during JAKi therapy. Active smoking, increased BMI, and pre-existing arterial hypertension emerged as significant predictors (HR 3.746, 9.853, and 1.219, respectively; *p* ≤ 0.05 for each; Table 3), emphasizing that the presence of chronic autoimmune rheumatic diseases does not overshadow the impact of traditional cardiovascular risk factors. Elevated UA and TC levels were also associated with an increased hazard ratio for non-major cardiovascular events, suggesting that beyond the alterations reflected by standard lipid parameters, there are other metabolic disorders that may contribute to residual risk.

These findings align with those provided by larger studies conducted on rheumatic patients, which showed that advanced age, obesity, arterial hypertension, and DM are major contributors to the increased incidence of cardiovascular events (myocardial infarction, stroke) [41]. This underscores that aggressive management of traditional cardiovascular risk factors remains essential even when patients receive targeted therapies such as JAKi. Our results also echo recent recommendations from the EULAR and the ESC guidelines [4,11], which advocate for systematic screening of cardiovascular risk factors in patients with inflammatory rheumatic diseases, regardless of the specific disease-modifying regimen employed. Our results have a major impact on clinical practice as they support the integration of routine lipid and UA monitoring, alongside vigilant screening for arterial hypertension and lifestyle factors, such as smoking, into the care protocol of patients receiving JAKi. Early identification and aggressive intervention on these cardiovascular risk factors may mitigate the residual cardiovascular burden in this vulnerable population.

Our results indicate that JAKi therapy may have a negative effect on the lipid profile. Increases in levels or lack of improvement in TC, LDL-C, and triglycerides were observed shortly after the initiation of JAKi therapy, persisting throughout the 12-month treatment period. Our results are in line with previous reports of lipid elevations during JAKi use [42,43,44]. This appears to be a class-wide effect of JAKi on lipid metabolism. Importantly, in our cohort, these lipid changes occurred despite 25% of patients being on lipid-lowering therapy. This highlights the need for vigilant cardiovascular risk management when initiating JAKi therapy, especially in patients with pre-existing comorbidities.

Previous studies indicate that statins remain effective in JAKi-treated patients, significantly mitigating drug-induced LDL-C and TC increases [45]. However, it is still unclear whether JAKi-induced lipid changes translate into higher rates of clinically overt cardiovascular events in the long term. It is conceivable that the anti-inflammatory benefits of JAKi could counterbalance some of the pro-atherogenic effects of lipid elevations. This hypothesis is supported by our results, namely a slight inverse correlation between “months on JAKi” and the presence of dyslipidemia (i.e., patients on JAKi were not more likely to meet dyslipidemia criteria after one year of therapy). This unexpected negative correlation may suggest a potentially beneficial effect of JAKi on lipid metabolism [42]. One possible explanation is that by reducing systemic inflammation, JAKi could improve lipid regulation or overall cardiovascular risk to an extent that mitigates the adverse impact of dyslipidemia on cardiovascular risk. However, the absence of stratification based on statin use or disease activity limits the interpretability of this finding. Further research is needed to determine the net effect of JAKi on cardiovascular risk after adjusting for concurrent lipid-lowering therapy and inflammation control.

We also explored the dynamics of Lp(a) over a 12-month period and its potential role as a predictor of non-major cardiovascular events. Our findings provide novel insight into Lp(a) behavior in the context of targeted immunomodulation, while reinforcing the central importance of traditional cardiovascular risk factors.

Lp(a) is an established independent risk factor for atherosclerosis. Still, its behavior under anti-rheumatic therapy has not been well studied [46]. To our knowledge, our pilot study is the first to evaluate Lp(a) levels in patients receiving JAKi. We observed a strong intra-individual correlation between baseline and 12-month Lp(a) levels, confirming that Lp(a) is highly stable over time. Despite the overall stability, paired tests revealed a small, but statistically significant shift in median Lp(a) levels after 12 months of JAKi therapy. This paradox of high correlation, yet slight change, suggests a subtle influence of treatment on Lp(a). One possible explanation is the reduction in systemic inflammation with JAKi therapy. Chronic inflammation is known to modulate Lp(a). The LPA gene contains IL-6 response elements, and Lp(a) behaves as an acute-phase reactant. High inflammatory states (such as rheumatic diseases) can drive Lp(a) upward, whereas effective cytokine blockade may relieve this stimulus [47]. Notably, Il-6 inhibition via tocilizumab has been shown to lower Lp(a) levels in RA patients [32]. JAKis partially attenuate IL-6 signaling, so a plausible interpretation is that dampening inflammation with JAKi therapy led to a slight decrease in Lp(a). This aligns with the direction of change we detected, although the magnitude was modest. It is important to note that prior short-term studies of tofacitinib in inflammatory disease reported no meaningful change in Lp(a) over 4 months [48], suggesting that any anti-inflammatory effect on Lp(a) is gradual or limited. Despite a minimal net change, the significant difference obtained in our study between baseline and 12-month assessment most likely reflects the sensitivity of paired comparisons even for minor shifts. Clinically, this finding implies that although controlling inflammation may slightly reduce Lp(a) from a high baseline value, it is insufficient to normalize Lp(a) when its levels are chiefly genetically determined. Our Cox analysis showed that elevated baseline Lp(a) levels, in combination with traditional cardiovascular risk factors such as arterial hypertension, smoking, or increased BMI, were associated with a higher hazard ratio of non-major cardiovascular events during follow-up. Interestingly, when post-treatment Lp(a) levels were considered, no significant associations with studied events were observed. One plausible explanation is that the anti-inflammatory effect of JAKi therapy might attenuate the pro-atherogenic impact of elevated Lp(a) over time, thereby reducing its short-term prognostic relevance.

Moreover, despite its stability, patients with abnormal (elevated) Lp(a) constituted a high-risk subgroup. In our cohort, those with elevated Lp(a) at baseline tended to have more obesity and arterial hypertension, and experienced more non-major cardiovascular events over time. However, future risk stratification models for RA/PsA patients on JAKi might be improved by incorporating Lp(a) alongside other biomarkers and considering patient age. Given the small sample size and lack of multivariate adjustment for confounders such as statin use or inflammatory burden, Lp(a) should currently be regarded as a hypothesis-generating biomarker, not a validated predictor. Larger, prospective studies with well-stratified cohorts are necessary to confirm its utility in this context. Future prediction models might benefit from integrating Lp(a) with additional biomarkers and demographic factors such as age. However, clinical decisions should not yet be based solely on Lp(a) levels in JAKi-treated patients.

Our study has several limitations. Firstly, the sample size was relatively small and drawn from a single center, which may limit the generalizability of the findings. Some results did not reach statistical significance due to limited power, and we could not rigorously compare the effects of different JAKi drugs (tofacitinib vs. baricitinib vs. upadacitinib) on cardiovascular outcomes. Secondly, the follow-up period of one year is relatively short for assessing major cardiovascular events, which often take many years to manifest; a longer observation period is necessary to evaluate the full impact of JAKi on cardiovascular health. The 12-month follow-up, while pragmatic, may be insufficient to capture the full impact of Lp(a) on cardiovascular outcomes, which typically accrue over years. Our composite of non-major cardiovascular events served as a surrogate for cardiovascular risk, but these outcomes vary in pathophysiology and may not fully represent atherosclerotic events. A longer observation period that would allow for obtaining hard endpoints, such as MI or stroke, would be informative. Thirdly, we did not include a control group of patients who did not receive JAKi. This observational before–after study design cannot definitively prove causation for the changes observed. To strengthen our analysis, each patient was used as their own control (baseline vs. follow-up). Furthermore, our regression models were adjusted for potential confounders such as age, sex, comorbidities, and concomitant statin use. Encouragingly, several of our findings (such as the predictors of cardiovascular events) mirror those of larger studies, supporting their validity despite the small sample size. Nevertheless, our study provides valuable exploratory assessment of Lp(a) behavior under JAKi treatment and reinforces the importance of implementing guideline recommendations for cardiovascular risk management.

Our pilot study generates hypotheses that warrant further investigation. Studies that include a larger number of patients, a control group, and a longer follow-up period are undeniably needed to confirm our results and determine whether certain therapies offer a more favorable cardiovascular risk profile. Although caution is warranted in interpreting our results for the time being, clinicians should continue to closely monitor cardiovascular risk factors in patients undergoing JAKi therapy. The main strength of our study is the evaluation of Lp(a) levels in patients receiving JAKi treatment. To our knowledge, this is the first study with this objective to date. Our findings suggest that incorporating novel biomarkers such as Lp(a) into risk assessment models (especially for younger patients) may facilitate early identification of individuals at high risk of future major cardiovascular events. In patients with multiple uncontrolled cardiovascular risk factors or very high Lp(a) levels, the decision to initiate JAKi should either be postponed until risk factors are under control or carefully weighed against alternative treatment options [10].

## 5. Conclusions

Our study provides preliminary evidence supporting the use of accessible biomarkers for cardiovascular risk stratification in patients receiving JAKi therapy. Although JAKi appears to influence lipid metabolism, Lp(a) levels showed strong intra-individual stability over time. Although a statistically significant change was detected, the absolute difference was small and likely of limited clinical relevance. This consistency, coupled with its known association with atherogenesis, positions Lp(a) as a potentially valuable biomarker for identifying those at high risk of future non-major cardiovascular events in patients with autoimmune rheumatic disease undergoing JAKi therapy. Larger cohorts, longer follow-up periods, and comparative studies are needed to validate and extend our findings.

## Figures and Tables

**Table 1:** Baseline clinical, serological and treatment characteristics of the study population.

Variable	*n* = 48
Demographic parameters	
Mean age (years)	56.83
Female/male ratio	4.3:1
Cardiovascular risk factors	
Current smoking, *n* (%)	8 (17%)
Increased BMI (kg/m^2^), *n* (%)	36 (75%)
Arterial hypertension, *n* (%)	28 (58%)
Dyslipidemia, *n* (%)	17 (35.4%)
Statin use, *n* (%)	12 (25%)
DM, *n* (%)	9 (19%)
PAD, *n* (%)	0
Arrythmias, *n* (%)	0
Chronic coronary syndrome	7 (14.6%)
History of stroke, *n* (%)	1 (2%)
History of MI, *n* (%)	0
Rheumatic disease characteristics	
Positive RF, *n* (%)	35 (76%)
Positive ACPA, *n* (%)	36 (78%)
JAKi therapy	
Baricitinib, *n* (%)	20 (42%)
Upadacitinib, *n* (%)	15 (31%)
Tofacitinib, *n* (%)	13 (27%)
Concomitant csDMARDs	
Methotrexate, *n* (%)	21 (44%)
Leflunomide, *n* (%)	16 (33%)
Sulfasalazine, *n* (%)	1 (2%)
Biological parameters	
Increased TC, *n* (%)	27 (56%)
Increased LDL-C, *n* (%)	27 (56%)
Increased triglycerides, *n* (%)	6 (12%)
Increased Lp(a), *n* (%)	9 (19%)
Increased UA, *n* (%)	7 (15%)

**Table 2:** Cardiovascular events recorded during the 12-month follow-up period in patients undergoing JAKi treatment.

Type of Event	Number of Events (*n*)	Number of Patients Affected	Method of Ascertainment
Uncontrolled arterial hypertension	18	18	BP measurement (BP ≥ 140/90 mmHg) at any visit
Stable angina pectoris	3	3	Clinical symptoms, ECG changes
Hospitalization for heart failure	0	0	Clinical symptoms
New-onset arrhythmia (e.g., AF)	2	2	ECG confirmation at any visit
MI	0	0	Clinical symptoms/ECG/TTE/biomarkers at any visit
Stroke	0	0	Clinical symptoms
Total	23	23	-

Legend: BP, blood pressure; ECG, electrocardiogram; AF, atrial fibrillation; MI, myocardial infarction. Events were documented prospectively and adjudicated by the treating medical team.

**Table 3:** Cox proportional hazard model for predictors of first non-major cardiovascular event.

*Cardiovascular Risk Factor*	HR	95% Confidence Interval (CI)	*p*-Value
**BMI ≥ 25 kg/m^2^**	**3.746**	**1.000–15.484**	**0.050**
**Active smoking**	**9.853**	**2.000–48.538**	**0.005**
**Arterial hypertension**	**1.219**	**1.061–1.793**	**0.021**

Abbreviations: HR, hazard ratio; CI, confidence interval; BMI, body mass index.

**Table 4:** Cox proportional hazard model evaluating biological biomarkers in relation to non-major cardiovascular events.

*Biological Parameter*	HR	95% CI	*p*-Value
**UA**	**1.515**	**1.018–2.255**	**0.040**
**TC**	**1.019**	**1.001–1.036**	**0.034**

Abbreviations: HR, hazard ratio; CI, confidence interval; UA, uric acid; TC, total cholesterol.

## Data Availability

The original contributions presented in this study are included in the article. Further inquiries can be directed to the corresponding author.
